# Blood-based biomarkers in hypothalamic-pituitary axes for the risk of dementia or cognitive decline: a systematic review and meta-analysis

**DOI:** 10.18632/aging.103813

**Published:** 2020-10-26

**Authors:** Yi-Jun Ge, Wei Xu, Chen-Chen Tan, Lan Tan

**Affiliations:** 1 Department of Neurology, Qingdao Municipal Hospital, Qingdao University, Qingdao, China

**Keywords:** blood biomarker, hormone, hypothalamic-pituitary, cognitive decline, dementia

## Abstract

Blood-based biomarkers are ideal candidates for dementia prediction. This systematic review and meta-analysis aimed to evaluate longitudinal relationships of blood hormones and hormone-binding proteins in hypothalamic-pituitary (HP) axes with dementia or cognitive decline. PubMed, MEDLINE, EMBASE, PsycINFO, and BIOSIS were systematically searched from 1919 to June 2020. Fifteen types of hormones and four types of hormone-binding proteins were measured in 48 prospective studies. Increased risk of dementia or cognitive decline could be predicted by elevated blood concentrations of free-thyroxine (free-T4, RR = 1.06, p = 0.001) and sex hormone-binding globulin (SHBG, RR = 1.10, p = 0.025). Lower thyroid-stimulating hormone (TSH) levels within (RR = 1.28, p < 0.001) and below (RR = 1.27, p = 0.004) the normal range were both risky. Current evidence suggests the alterations of multiple blood molecules in HP axes, especially TSH, free-T4, and SHBG precede the incidence of dementia or cognitive decline. The underpinning etiology remains to be elucidated in the future.

## INTRODUCTION

Dementia, with cognitive impairment as the core symptom, has brought tremendous burden on health and social care in the 21^st^ century [1]. Aging is accompanied by an increased prevalence of dementia, as well as an altered blood concentration of multiple hormones and hormone-binding proteins [2]. However, it remains disputable whether the hormonal change is involved in the occurrence of cognitive disorders, or it is merely part of the normal aging progress. If the hormonal changes in blood could predict cognitive deterioration, the blood tests of hormones might be greatly valuable in screening and identifying individuals at high risk of developing dementia, especially considering that they are less invasive and more accessible than other approaches, such as cerebrospinal fluid and PET imaging [3].

In past decades, lines of evidence suggested that the dysregulation of hypothalamic-pituitary-thyroid (HPT) [4], -gonadal (HPG) [5], -somatic (HPS) [6], and -adrenal (HPA) [7] axes was linked to cognitive impairments. The homeostasis disturbance might be caused by the aberrant metabolism of hormones *per se* or changed bioavailable fractions due to abnormal levels of hormone-binding proteins [8]. Higher brain amyloid burden [9] and greater brain atrophy [10] were detected in individuals who had alterations in hormone levels, providing insight into pathophysiological mechanisms. Still, the risk for dementia and cognitive decline caused by hormonal changes was under debate, which might be attributed to different study designs, inconsistent outcome criteria, and mixed fractions of hormones. For instance, testosterone and estradiol were found to impart no or detrimental effects on cognition in recent meta-analyses of randomized controlled trials (RCTs) [11, 12]. These findings posed a challenge to the traditional view that they were both neuroprotective [8, 13]. To date, most studies in this topic only select certain types of hormones instead of exploring the whole hypothalamic-pituitary (HP) axes. It should be noted that these axes share common anatomical basis and similar negative feedback loops, suggesting potential common pathophysiological pathways to cognitive deterioration.

Herein, we aimed to evaluate the relationships of hormones and hormone-binding proteins in HP axes with all-cause dementia and cognitive decline based on prospective studies.

## RESULTS

### Study selection and characteristics

**Figure 1:**
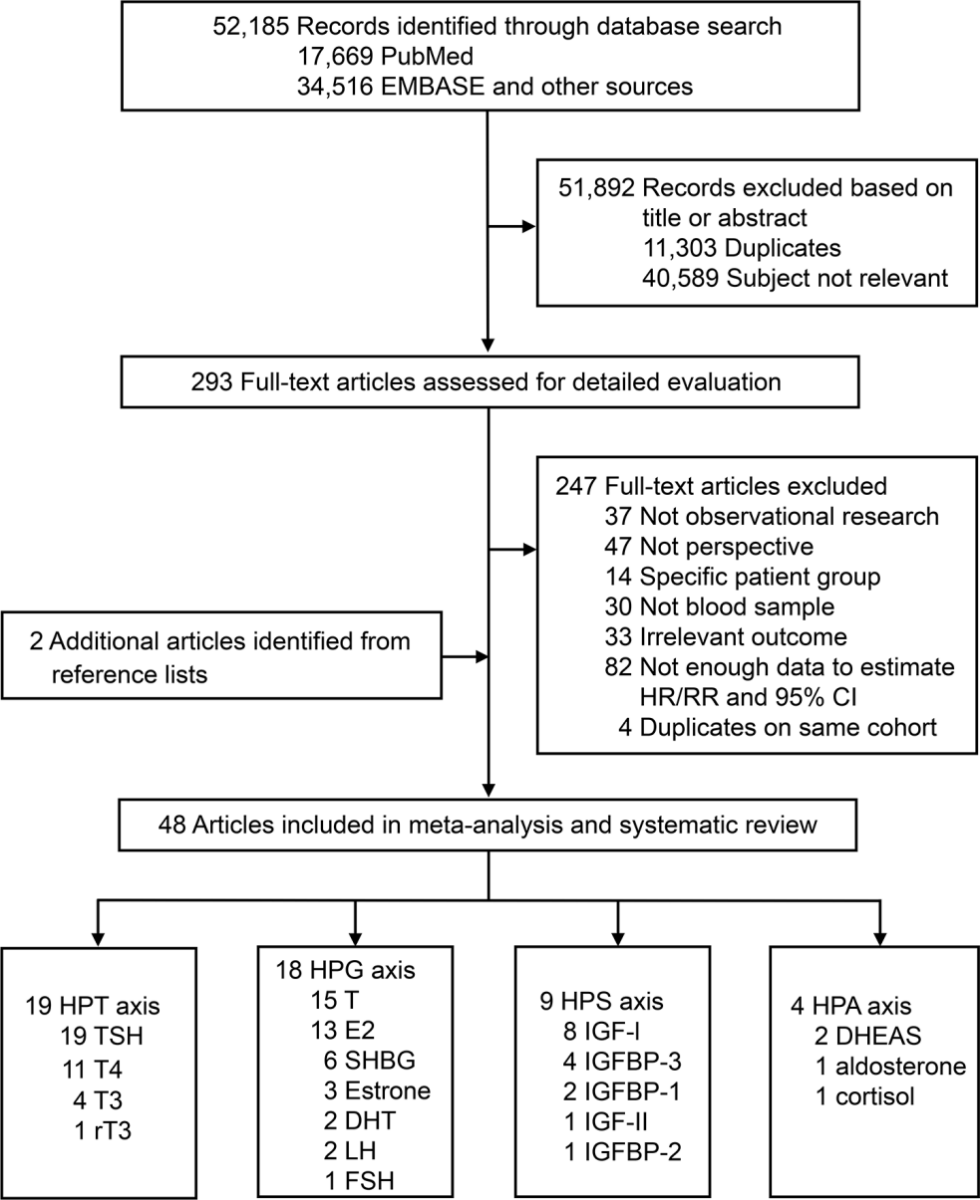
**Literature search for the systematic review and meta-analysis.** Abbreviations: DHEAS, dehydroepiandrosterone sulfate; DHT, dihydrotestosterone; E2, estradiol; FSH, follicle-stimulating hormone; GH, growth hormone; HPA, hypothalamic-pituitary-adrenal; HPG, hypothalamic-pituitary-gonadal; HPT, hypothalamic-pituitary-thyroid; HPS, hypothalamic-pituitary-somatic; IGF, insulin-like growth factor; IGFBP, insulin-like growth factor-binding protein; LH, luteinizing hormone; rT3, reverse triiodothyronine; SHBG, sex hormone-binding globulin; T, testosterone; T3, triiodothyronine; T4, thyroxine; TSH, thyroid-stimulating hormone.

Figure 1 summarizes the selection process. In brief, the search strategy returned 52,185 records, from which we excluded 51,892 after screening the title or abstract, leaving 293 potentially eligible publications. After reviewing the full-text, 247 articles were further excluded. In addition, two eligible articles were identified through screening of reference lists. Finally, a total of 48 articles were included in the systematic review and meta-analysis, including one with unpublished data.

Table 1 exhibits the study characteristics of all included studies. In total, 15 types of hormones and 4 types of hormone-binding proteins involving HPT, HPG, HPS and HPA axes were systematically reviewed. Hormones identified were thyroid-stimulating hormone (TSH) [10, 14–31], thyroxine (T4) [10, 17–21, 23, 26–28, 30], triiodothyronine (T3) [10, 17, 23, 28], and reverse triiodothyronine (rT3) [10] in HPT axis; luteinizing hormone (LH) [32, 33], follicle-stimulating hormone (FSH) [32], testosterone (T) [8, 32–45], dihydrotestosterone (DHT) [32, 33], estrone (E1) [32, 41, 46], and estradiol (E2) [8, 32–34, 36, 38, 39, 41, 43, 45–48] in HPG axis; insulin-like growth factor (IGF) -I [49–56], and IGF-II [51] in HPS axis; dehydroepiandrosterone sulfate (DHEAS) [42, 57], aldosterone [55] and cortisol [58] in HPA axis. Hormone-binding proteins identified were sex hormone-binding globulin (SHBG) [32, 33, 37–40] in HPG axis; insulin-like growth factor-binding protein (IGFBP)-1 [52, 56] IGFBP-2 [59] and IGFBP-3 [49, 51, 52, 56] in HPS axis.

**Table 1:** Characteristics of prospective studies included in the systematic review and meta-analysis.

**Study**	**Mean age; female**	**Biomarker**	**Sample for analysis; observation**	**Main outcome**
**Hypothalamic-pituitary-thyroid (HPT) axis**				
Annerbo et al., 2006	65.0; 52%	TSH (across)	93; 6y	AD
Annerbo et al., 2009	81.0; 75%	TSH (across)	200; 6.7y	AD
Aubert et al., 2017	75.1; 52%	low TSH, high TSH	2,251; 9y	dementia
Cappola et al., 2015	74.5; 56%	TSH (within), free-T4, total-T3	1,824; 17y	dementia
Castellano et al., 2013	75.9; 29%	TSH (within), total-T4, free-T4, total-T3, free-T3	62; 3y	cognitive decline
Chaker et al., 2016	64.9; 57%	TSH (across), TSH (within), free-T4	7,966; 8y	dementia
Dejong et al., 2006	72.3; 51%	TSH (across), low TSH, high TSH, free-T4, total-T3, rT3	1,025; 5.5y	dementia, AD
Dejong et al., 2009	78.1; 0%	TSH (across), low TSH, high TSH, total-T4, free-T4	665; 4.7y	dementia, AD
Folkestad et al., 2020	63; 77%	low TSH	13,445; 7.3y	dementia
Forti et al., 2012	73.3; 53%	TSH (across), high TSH, free-T4	660; 3.8y	dementia and its subtypes
George et al., 2019	57; 56%	TSH (across), low TSH, high TSH, free-T4	12,481; 21.9y	dementia
Hogervorst et al., 2008	73.6; 51%	TSH (across), free-T4	899; 2y	cognitive decline
Kalmijn et al., 2000	68.8; 72%	low TSH, high TSH	1,730; 2.1y	dementia, AD
Moon et al., 2014	72.5; 50%	TSH (within)	313; 5y	MCI or dementia
Quinlan et al., 2019	65.0; 54%	TSH (across), free-T4, free-T3	302; 2.8y	dementia and its subtypes
Tan et al., 2008	71; 59%	TSH (across), TSH (within)	1,600; 12.7y	dementia, AD
Vadiveloo et al., 2011	66.5; 77%	low TSH	12,115; 5.6y	dementia
Volpato et al., 2002	77.2; 100%	TSH (within), total-T4	464; 3y	cognitive decline
Yeap et al., 2012	78.3; 0%	TSH (across), TSH (within), high TSH, free-T4	3,401; 5.9y	dementia
**Hypothalamic-pituitary-gonadal (HPG) axis**				
Carcaillon et al., 2014	74.5; 0%	total-T, bio-T, total-E2	503; 3.1y	dementia and its subtypes
Carcaillon et al., 2014	75.2; 100%	total-T, total-E2, bio-E2	675; 4y	dementia, AD
Chu et al., 2010	72.7; 0%	bio-T	153; 1y	dementia, AD
Ford et al., 2018	77.0; 0%	LH, total-T, free-T, DHT, total-E2, SHBG	4,069; 10.5y	dementia
Geerlings et al., 2003	69.8; 54%	total-E2, bio-E2	1,031; 6.3y	dementia and its subtypes
Geerlings et al., 2006	77.4; 0%	bio-T, bio-E2	2,300; 6.1y	dementia and its subtypes
Hogervorst et al., 2010	74; 0%	total-T, free-T, SHBG	240; 2y	cognitive decline
Hsu et al., 2015*	76.9; 0%	FSH, LH, total-T, free-T, DHT, estrone, total-E2, SHBG	546; 5y	cognitive decline
Laughlin et al., 2010	69.0; 100%	estrone, total-E2, bio-E2	343; 4y	cognitive decline
LeBlanc et al., 2010	73.6; 0%	free-T, free-E2, SHBG	1,001; 4.5y	cognitive decline
Moffat et al., 2004	66.3; 0%	total-T, free-T, SHBG	574; 19.1y	dementia, AD
Muller et al., 2009	77.4; 0%	total-T, free-T, estrone, total-E2, free-E2	218; 4y	cognitive decline
Muller et al., 2010	77.4; 70%	SHBG	731; 5.2y	dementia, AD
Ponholzer et al., 2009**	75.7; 0%	total-T	146; 5y	AD
Ravaglia et al., 2007	73.9; 54%	free-T, total-E2	809; 3.8y	dementia and its subtypes
Suravarapu et al., 2006	72.7; 0%	total-T, bio-T	128; 10.3y	dementia
Yaffe et al., 2000	71.4; 100%	total-T, free-T, bio-E2, free-E2	292; 6y	cognitive decline
Yaffe et al., 2007	75.2; 45%	bio-E2	736; 2y	cognitive decline
**Hypothalamic-pituitary-somatic (HPS) axis**				
Almeida et al., 2017	76.9; 0%	total IGF-I, IGFBP-3	3,432; 9.2y	dementia
Dik et al., 2003	75.5; 51%	total IGF-I	1,022; 3y	cognitive decline
Green et al., 2014	56.1; 0%	total IGF-I, bio IGF-I, total IGF-II, IGFBP-3	745; 17y	dementia
Kalmijn et al., 2000	67.4; 50%	total IGF-I, bio IGF-I, free IGF-I, IGFBP-1, IGFBP-3	166; 1.9y	cognitive decline
McGrath et al., 2019	68.7; 53%	IGFBP-2	1,596; 11.8y	dementia, AD
Paulsen et al., 2019***	66.9; 59%	total IGF-I	970; 5y	MCI or dementia
Quinlan et al., 2017	64.6; 57%	total IGF-I	342; 3.6y	dementia and its subtypes
Westwood et al., 2014	65.0; 57%	total IGF-I	3,582; 7.4y	dementia, AD
Zhang et al., 2020	76.1; 55%	total IGF-I, bio IGF-I, IGFBP-1, IGFBP-3	655; 6.9y	MDCI
**Hypothalamic-pituitary-adrenal (HPA) axis**				
Kalmijn et al., 1998	67.1; 50%	DHEAS	169; 1.9y	cognitive decline
Paulsen et al., 2019***	66.9; 59%	aldosterone	970; 5y	MCI or dementia
Ponholzer et al., 2009**	75.7; 0%	DHEAS	146; 5y	AD
Schrijvers et al., 2011	72.0; 38%	cortisol	3,341; 7.1y	dementia, AD

Abridged table. Please see online Supplementary Table 1 for more detailed information.

*Unpublished data acquired by contacting with authors. **An overlapped article concerning HPG and HPA axes. ***An overlapped article concerning HPA and HPS axes. Abbreviations: AD, Alzheimer’s disease; bio, bioavailable; DHEAS, dehydroepiandrosterone sulfate; DHT, dihydrotestosterone; E2, estradiol; FSH, follicle-stimulating hormone; high TSH, TSH level above normal range; IGF, insulin-like growth factor; IGFBP, insulin-like growth factor-binding protein; LH, luteinizing hormone; low TSH, TSH level below normal range; MCI, mild cognitive impairment; MDCI, multiple-domain cognitive impairment; rT3, reverse triiodothyronine; SHBG, sex hormone-binding globulin; T, testosterone; T3, triiodothyronine; T4, thyroxine; TSH, thyroid-stimulating hormone; TSH (across), TSH level across normal range; TSH (within), TSH level within normal range.

Out of the 48 eligible articles, 45 were community or population-based, and the other three studies were composed of patients with mild cognitive impairment (MCI) recruited from memory clinics. Total sample size of each axis ranged from 4,626 (HPA axis) to 61,496 (HPT axis). The mean ages of participants in all four axes were above 65 years at baseline, so samples in this review represent predominantly older people. Females were a majority of HPT (62%) axis, a minority of HPG (22%) and HPS (38%) axes and accounted for almost half in HPA (56%) axis. Gender differences in these axes were discussed below. Most studies were performed in western countries, except two in Asia [29, 35]. About half of the studies (22 out of 48) described they used morning fasting blood samples. The mean follow-up period of all included studies ranged from 1 to 21.9 years (median, 5.1 years). The mean quality score of each biomarker ranged from 6 to 9 stars, suggesting the overall quality was good.

### Hypothalamic-pituitary-thyroid (HPT) axis

**Figure 2:**
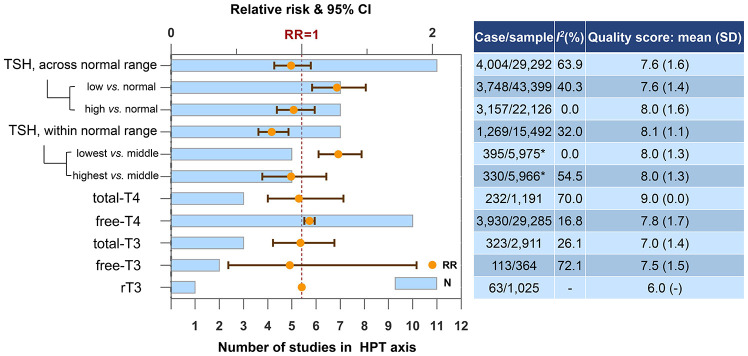
**Higher concentrations of blood biomarkers in HPT axis and the risk of dementia or cognitive decline.** *Only one of the five studies reported case and sample size. Abbreviations: rT3, reverse triiodothyronine; T3, triiodothyronine; T4, thyroxine; TSH, thyroid-stimulating hormone.

TSH and free-T4 levels were associated with the risk of dementia or cognitive decline (Figure 2). Higher TSH within the normal range represented a lower risk of dementia or cognitive decline (N=7, RR=0.77, 95% CI=0.67-0.90, *I^2^*=32.0%). However, the statistical significance was lost when meta-analyzing TSH level across the normal range. In additional analysis, we further proved that it was low but not high TSH level was associated with an increased risk of dementia or cognitive decline, regardless the normal range (below normal range *vs.* normal rage: N=7, RR=1.27, 95% CI=1.08-1.49, *I^2^*=40.3%; lowest *vs.* middle quantile within the normal rage: N=5, RR=1.28, 95% CI=1.13-1.46, *I^2^*=0.0%). Higher free-T4 (the unbound T4 in circulation) also increased the risk of dementia or cognitive decline (N=10, RR=1.06, 95% CI=1.02-1.10, *I^2^*=16.8%). Four studies provided gender-specific data. Consistent findings were high free-T4 was detrimental to cognition in male [19, 27] while TSH level was not associated with the risk in male [19, 24, 27]. The intermediate concentration of TSH in female tended to have the lowest risk of dementia or cognitive decline [24, 26]. Egger's test did not indicate any evidence of publication bias regarding TSH across the normal range (p=0.39) or free-T4 (p=0.07).

### Hypothalamic-pituitary-gonadal (HPG) axis

**Figure 3:**
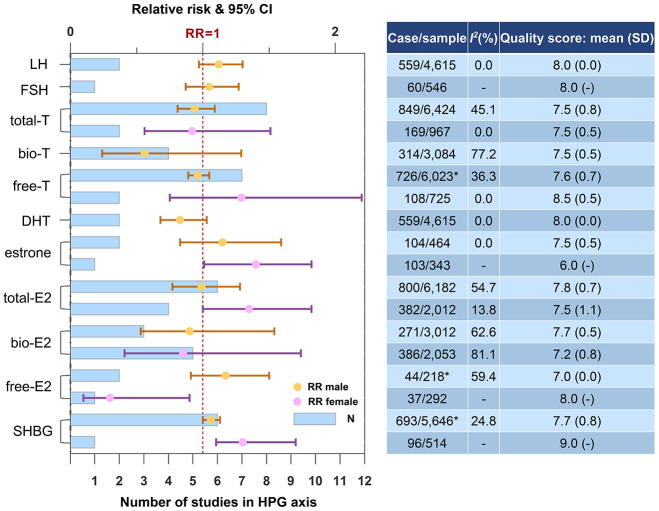
**Higher concentrations of blood biomarkers in HPG axis and the risk of dementia or cognitive decline.** *One of the studies did not report case and sample size. Abbreviations: bio, bioavailable; DHT, dihydrotestosterone; E2, estradiol; FSH, follicle-stimulating hormone; LH, luteinizing hormone; SHBG, sex hormone-binding globulin; T, testosterone.

Figure 3 shows the results in HPG axis stratified by gender. Higher SHBG increased the risk of dementia or cognitive decline in male (N=6, RR=1.06, 95% CI=1.00-1.13, *I^2^*=24.8%), female (N=1, RR=1.30, 95% CI=1.10-1.70) and both genders (N=7, RR=1.10, 95% CI=1.01-1.20, *I^2^*=46.4%). Gender differences were observed regarding the effect of gonadal hormones. In female, higher total-E2 was risky (N=4, RR=1.35, 95% CI=1.00-1.82, *I^2^*=13.8%), while bio-E2 and free-E2 were not. Higher estrone was also risky in female (N=1, RR=1.40, 95% CI=1.01-1.82). In male, none of the androgens was associated with dementia or cognitive decline, though a protective tendency had been detected. Neither estrogens in male nor androgens in female reached statistical significance.

### Hypothalamic-pituitary-somatic (HPS) axis

**Figure 4:**
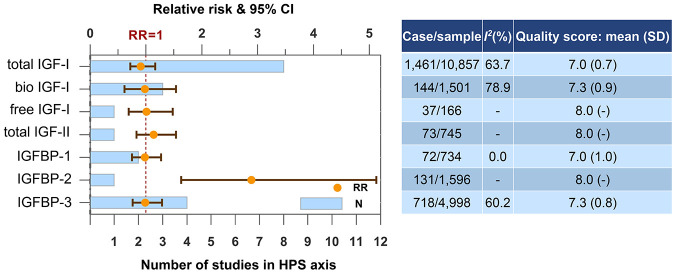
**Higher concentrations of blood biomarkers in HPS axis and the risk of dementia or cognitive decline.** Abbreviations: bio, bioavailable; IGF, insulin-like growth factor; IGFBP, insulin-like growth factor-binding protein.

IGFBP-2, the most predominant IGFBP in the brain, is the only blood biomarker in HPS axis associated with dementia risk (Figure 4). Result from the Framingham Heart Study Offspring cohort showed elevated circulating IGFBP-2 levels increased the dementia risk with a relatively large effect size (RR=2.89, 95% CI=1.63-5.13). We conducted a subgroup analysis regarding IGF-I according to gender and did not find gender difference.

### Hypothalamic-pituitary-adrenal (HPA) axis

**Figure 5:**
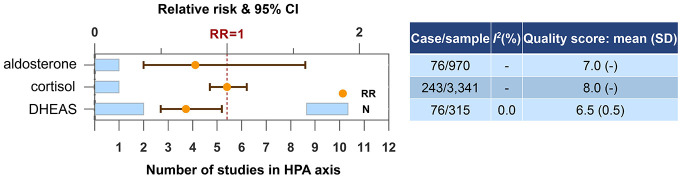
**Higher concentrations of blood biomarkers in HPA axis and the risk of dementia or cognitive decline.** Abbreviation: DHEAS, dehydroepiandrosterone sulfate.

The number of studies and participants regarding blood molecules in HPA axis was limited (Figure 5). Only DHEAS was found to be a candidate biomarker for the risk of dementia or cognitive decline. Higher blood level of DHEAS was protective (N=2, RR=0.69, 95% CI=0.50-0.96, *I^2^*=0.0%).

## DISCUSSION

**Figure 6:**
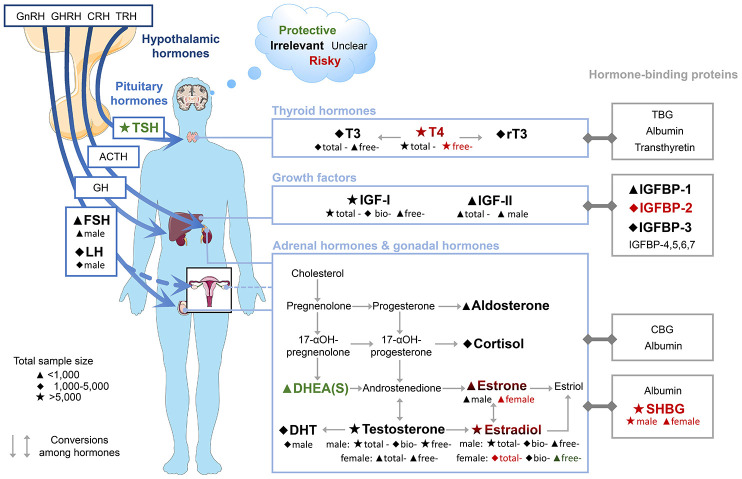
**Map of hormones in HP axes, their hormone-binding proteins and the risk of dementia and cognitive decline.** Font colours indicate the effect on risk of dementia or cognitive decline of higher concentrations of blood biomarkers. Subgroups are shown below biomarker names. The results of FSH, LH, and IGF-II were derived from males only. Abbreviations: ACTH, adrenocorticotropic hormone; bio, bioavailable; CBG, corticosteroid-binding globulin; CRH, corticotropin-releasing hormone; DHEAS, dehydroepiandrosterone sulfate; DHT, dihydrotestosterone; FSH, follicle-stimulating hormone; GH, growth hormone; GHRH, growth hormone-releasing hormone; GnRH, gonadotropin-releasing hormone; IGF, insulin-like growth factor; IGFBP, insulin-like growth factor-binding protein; LH, luteinizing hormone; rT3, reverse triiodothyronine; SHBG, sex hormone-binding globulin; T3, triiodothyronine; T4, thyroxine; TBG, thyroxine-binding globulin; TRH, thyrotropin-releasing hormone; TSH, thyroid-stimulating hormone.

In this study, we found the increased risk of dementia or cognitive decline could be predicted by a dropped blood concentration of TSH or DHEAS, as well as an elevated blood concentration of free-T4, SHBG, or IGFBP-2. In subgroup analysis, elevated levels estrogen (total-E2 and estrone) in female were also associated with the high risk. Among these blood-based biomarkers, evidence of three (TSH, free-T4 and SHBG) was robust on the basis of the large sample size (>5,000), consistency (*I^2^* <50%), and="" high="" quality="" (NOS=""<7). Figure 6 shows a map of hormones in HP axes, their hormone-binding proteins, and the risk for dementia or cognitive decline.

Low TSH and high free-T4 indicate hyperthyroidism and were both detrimental to cognition in our meta-analysis. It had been demonstrated that such a distribution of hormone levels was linked to multiple adverse outcomes, including atrial fibrillation and heart failure [17], some of which were established risk factors for dementia. Hence, the increased risk of dementia or cognitive decline might be explained by the cardiovascular side effects to a certain extent.

All eligible studies on total-E2 in female excluded hormone therapy users, so they represented the endogenous total-E2. The detrimental effect of endogenous high total-E2 in female was consistent with a meta-analysis of RCTs that reported exogenous estrogen plus progestin therapy increased the risk of probable dementia in female [11]. It has been suggested that estrogens were neuroprotective for younger postmenopausal women while neurotoxic for older women [60]. Females in eligible studies we included for this topic were old (mean age, 69-75 years), so our data supported this hypothesis. The dementia risk of total-E2 in female could also be explained by the free hormone hypothesis, as we observed bioavailable E2 failed to predict the risk, while high SHBG (the predominant E2-binding protein in circulation) was associated with higher risk of dementia. Consistently, we recently reported that higher levels of plasma SHBG contributed to accelerated Alzheimer’s pathology, declined brain metabolism, and atrophy of the hippocampus, which were known to be involved in the pathophysiology of cognitive decline [61]. The null finding of endogenous androgens in male was in line with a recent evidence-based guideline, indicating exogenous testosterone therapy in older men had little to no effect on cognition [62].

Taken together, the alteration in blood concentration of multiple hormones and hormone-binding proteins in HP axes precedes the incidence of dementia and cognitive decline, though the related etiology is a subject of ongoing research and debate. Interestingly, high SHBG is generally accompanied by low TSH, high free-T4, and high estrogen [63], and they were all associated with a high risk of dementia or cognitive decline in our meta-analysis. This linkage suggests the risk for dementia and cognitive decline is not merely caused by some individual components, but the whole HP axes is involved. The hypothesis was supported by other pathological and neuroimaging studies [64]. Hypothalamic neuronal loss, atrophy and metabolism reduction were detected in patients with neurodegenerative diseases compared to healthy controls. Hypothalamic dysfunctions, including disrupted regulation of body weight, circadian rhythm and sleep were also common in patients with dementia.

Admittedly, effect sizes of most biomarkers in this review were relatively small, but still valuable for dementia prediction, especially in primary care setting or large-scale screening. There is a high prevalence of thyroid disease in the elderly population, and many apparently healthy individuals receive a routine examination of thyroid function [4]. Meanwhile, plasma SHBG has been applied to clinical examinations of various metabolic diseases. Therefore, we recommend greater attention be paid to cognitive besides metabolic effects when the concentration of these biomarkers in blood deviates from normal. These results also provided potential therapeutic targets for dementia. Of note, our findings were based on the population level and thus could not be extended directly to an individual level. Future studies for accurate prediction of cognitive. decline at the individual level should consider age, gender, comorbidities, and the measurement techniques. In our subgroup analysis, gender differences were observed regarding the effect of several biomarkers on cognition. This could be explained by natural differences in hormone concentrations of both genders. The prevalence of dementia phenotypes among men and women differs [65], which could also be a possible explanation.

Our study has several limitations. First, although a total of 48 studies were included, not all of them covered the same biomarkers. For most biomarkers, the actual number of studies was insufficient to draw a firm conclusion, so the results should be interpreted with caution and warrant replication in more prospective cohorts. Likewise, subtypes of dementia were unable to be distinguished due to the limited number of studies. Second, only studies with RR and 95% CI were included, so some valuable information derived from different statistical methods might be omitted. Third, the U-shaped association between certain hormones and dementia risk had been suggested [8, 24]. However, we could not conduct the dose-response analysis to test the hypothesis because of the heterogeneous units and reference levels among different laboratories. Fourth, multiple factors are associated with the concentration of hormones, and hormones themselves follow complex regulatory pathways. Although maximally adjusted estimates were applied, over- or under-adjustment may exist and cofound the results. Fifth, diurnal change is an important consideration to develop blood-based biomarkers. We had no means to further investigate this issue for half studies in the meta-analysis did not control this aspect sufficiently.

Despite these caveats, this study had a number of strengths. First, it was comprehensive. We explored hormones and hormone-binding proteins in HP axes as wildly as possible to find the potential connection of biomarkers in different axes for their impact on cognition. Second, as only prospective studies were included, we were able to predict the risk of dementia and cognitive decline before the disease onset. Third, the participants included in our study were mainly community-dwelling elders selected from multiple sampling, so our findings could provide recommendations for this specific population.

In conclusion, we found the endocrine profile of low TSH, high free-T4 and high SHBG was associated with an elevated risk of dementia or cognitive decline. The underpinning etiology remains to be elucidated. Future efforts were needed to develop individualized blood-based models for predicting dementia and cognitive decline using multiple elements, including hormones and hormone-binding proteins.

## MATERIALS AND METHODS

This systematic review and meta-analysis was prepared following recommendations of the Meta-analysis Of Observational Studies in Epidemiology (MOOSE) and the Preferred Reporting Items for Systematic Reviews and Meta-Analyses (PRISMA) groups [66, 67].

### Search strategy

We systematically searched PubMed, MEDLINE, EMBASE, PsycINFO and BIOSIS from 1919 to June 29, 2020 without language restriction, using the search terms “hormone”, “thyroid stimulating hormone”, “TSH”, “thyrotropin”, “thyroxine”, “triiodothyronine”, “hypothyroidism”, “hyperthyroidism”, “thyroid”, “follicle stimulating hormone”, “FSH”, “luteinizing hormone”, “LH”, “lactogen”, “pituitary”, “sex hormone”, “androgen”, “testosterone”, “dihydrotestosterone”, “DHT”, “estrogen”, “estradiol”, “estriol”, “estrone”, “progesterone”, “sex hormone binding globulin”, “SHBG”, “growth hormone”, “GH”, “insulin like growth factor”, “IGF”, “corticotropin”, “adrenocorticotropic hormone”, “ACTH”, “dehydroepiandrosterone, “DHEA”, “dehydroepiandrosterone sulfate”, “DHEAS”, “cortisol”, “adrenal”, “dementia”, “Alzheimer”, “cognitive” and “MCI”. The reference lists of eligible articles and relevant reviews were hand-searched for additional citations.

### Selection criteria

Eligible studies had to meet the following criteria simultaneously: (1) had a prospective design (prospective cohort, case-cohort or nested case-control); (2) participants were selected from general populations or memory clinic without dementia; (3) dementia or cognitive decline were recorded by use of well-defined criteria; (4) relative risks (RRs) and 95% confidence intervals (CIs) regarding the association between endogenous blood hormones or hormone-binding proteins in HP axes and dementia or cognitive decline were initially given or could be calculated by information derived from articles. Studies were excluded if they failed to meet any criteria described above. Studies on hormone replacement therapy were not included because this topic had been investigated in recent systematic reviews [11, 12]. If a cohort related to the same biomarker had been published more than once, we chose the most recent publication. The eligibility of each study was assessed by two investigators independently. Disagreements regarding eligibility were resolved by consensus.

### Data extraction

The following information was extracted: first author, publication year, study design, cohort name, source, country, duration of follow-up, follow-up rate, demographic characteristics (age, gender, case and sample size, whether dementia and hormonal medication users were excluded at baseline), exposure (type of biomarkers, whether fasting morning blood sample was collected, hormone measurement technique, coefficients of variation), and outcome (dementia or cognitive decline and related diagnostic criteria, confounders adjusted). The maximally adjusted estimate was applied when several adjusted models were reported in one study. We contacted corresponding authors for further explanation regarding uncertain and missing data. Two investigators independently extracted data. Disagreements were settled by consensus with the help of a third investigator.

### Quality assessment

The Newcastle-Ottawa Scale was used to assess the methodological quality of the included studies. The scale evaluates three domains: selection, comparability, and outcome ascertainment. In this systematic review, we graded the quality as high (7-9 stars), moderate (4-6 stars), and poor (1-3 stars).

### Statistical analysis

Risk estimates were expressed as RRs with 95% CIs. A RR above one indicates the high level in blood increases the risk of dementia or cognitive decline, while a RR below one refers to the low level increases the risk. Hazard ratios (HRs) were deemed to be equivalent to RRs. As odds ratios (ORs) often overestimate the true effect, we converted them to RRs using the following formula [68]:

$$RR_{adjusted}=\;OR_{adjusted}/[\left(1-P_{0}\right)+(P_{0}\times OR_{adjusted})]$$

P_0_ indicates the incidence of outcome in the reference group or the total sample (when the former is not available). If feasible, RRs and 95% CIs were estimated manually by raw data derived from the text when the original data was not available. We combined the continuous data (*i.e.*, RR per unit increment) with categorical data (*i.e*., RR of highest *vs.* lowest quantile), and we chose the latter in priority when they were both provided in the same study. For categorical data, if original risk estimates were not presented as the top versus bottom quantile, we graded the bottom quantile as the reference level and recalculated the risk estimate based on a proposed method [69]. The units of hormone levels were not normalized due to the heterogeneous measurement approaches among studies.

The free or bioavailable hormones cross the blood-brain-barrier more readily and may be better correlated with cognition [48]. Since some hormones have been reported as different fractions according to bioavailability, we pooled them separately. For those bioavailable fractions, direct laboratory measurements and prediction formulas estimating their blood concentrations were combined. As the normal range of TSH was available in most studies, we defined its four categories based on the normal range: across the normal range, within the normal range, above the normal range (high TSH) and below the normal range (low TSH). Additional analysis was conducted to further investigate the primary results of TSH, using four comparisons: below normal range *vs.* normal range, above normal range *vs.* normal range, the lowest *vs.* the middle quantile within the normal range, the highest *vs.* the middle quantile within the normal range. Prespecified subgroup analysis according to gender in HPG axis was conducted in case of the possible sex difference.

Heterogeneities between studies were assessed by the *I^2^* statistic. *I^2^* values of 25%, 50%, and 75% represent possibly low, moderate, and high heterogeneity, respectively. Publication bias was evaluated by the Egger's test if there were at least 10 eligible studies. RRs and 95% CIs were log-transformed and pooled using random-effects models. If a study reported data only on Alzheimer’s disease (AD) and vascular dementia (VD) but no data on all-cause dementia, we used the fixed effects model to calculate its RR and 95% CI to estimate the risk of all-cause dementia before the main synthesis. Dementia was preferred if data on dementia and cognitive decline were both available for the same biomarker measured in the same population. Analyses were conducted using Stata version 12.0 and R version 3.5.2.

## Supplementary Material

Supplementary Table 1
